# TOWARD AGE-FRIENDLY WORKPLACES: IDENTIFYING FACTORS THAT SUPPORT COGNITIVE FUNCTION IN OLDER WORKERS

**DOI:** 10.1093/geroni/igac059.2506

**Published:** 2022-12-20

**Authors:** Ashley Price, Soomi Lee

**Affiliations:** University of South Florida-Tampa, Tampa, Florida, United States; University of South Florida, Tampa, Florida, United States

## Abstract

Older adults in the workforce face natural age-related decline that may impede their work performance. Sleep and cognitive function, both of which are degraded with age, may affect work performance in older workers. Workplace demands and support may also play roles in older workers’ performance. Yet there remains a lack of effort in identifying modifiable factors that contribute to older workers’ performance. This review compiled previous studies on modifiable factors across personal and workplace domains that support or impede older workers’ performance at work. Databases utilized for this systematic review include Google Scholar, AgeLine, and APA PsycINFO. Inclusion criteria were empirical studies conducted in developed countries, published in 2000 or later, that focused on older adults (age >55) working full-time (≥ 35 hours/week). Keywords included: sleep, older adults, workforce, cognition, aging, work performance, aging workforce, workplace support, management strategies. Of the 32 studies initially identified, 13 qualified for this analysis. In 6 studies, poorer sleep (measured by actigraphy) was prevalent in older workers and was negatively associated with their cognitive performance at work. Across 7 studies, demanding and non-supportive workplace characteristics (i.e., greater job demands, lower supervisor support, and higher agism) were identified as common risk factors for poorer sleep, poorer cognitive function, and lower work performance in older workers. Bridging this information together may help identify specific factors that may be modifiable by workplace interventions to support optimal performance in workers and promote more age-friendly work environments.

